# Premonitory Symptoms of Migraine in Childhood and Adolescence

**DOI:** 10.1007/s11916-017-0631-y

**Published:** 2017-06-24

**Authors:** N. Karsan, P. Prabhakar, P. J. Goadsby

**Affiliations:** 1 NIHR-Wellcome Trust King’s Clinical Research Facility, King’s College Hospital, Denmark Hill, London, SE5 9PJ UK; 2 Department of Basic and Clinical Neuroscience, Institute of Psychiatry, Psychology and Neuroscience, Kings College London, Denmark Hill, London, SE5 9PJ UK; 3 Great Ormond Street Hospital for Children, Great Ormond St, London, WC1N 3JH UK

**Keywords:** Migraine, Paediatric, Adolescent, Premonitory, Prodrome

## Abstract

**Purpose of Review:**

Premonitory symptoms in migraine; symptoms occurring before the onset of migraine pain or aura, are an increasingly recognised area of interest within headache research. It has been recently documented in the literature that these symptoms also occur in children and adolescents, with a comparable phenotype to adults. This review discusses the wide presentation of premonitory symptoms in migraine in children and adolescents, and the importance of understanding how these early symptoms are mediated in order to ensure that targeted abortive therapies are developed in the future. Recognition of these symptoms by parents, guardians, teachers and carers is of importance in ensuring early and effective attack treatment.

**Recent Findings:**

A previous clinic-based questionnaire study in 103 children found a prevalence of premonitory symptoms in paediatric migraine of 67%, with a mean number of reported symptoms of two. A recent study found that in a clinic population of 100 children or adolescents with a migraine diagnosis who were preselected as having at least one premonitory symptom associated with their attacks, two or more premonitory symptoms were reported by 85% of patients. The most common symptoms were fatigue, mood change and neck stiffness.

**Summary:**

Although the population prevalence of premonitory symptoms in migraine within the paediatric population, or their ability to predict accurately the onset of an impending headache cannot be deduced from the retrospective studies performed to date, premonitory symptoms occur in children as young as 18 months old. Understanding the biological basis of these, and their heterogeneous phenotype may help future targeted therapeutic research, helping the development of drugs that act before the onset of pain, limiting the morbidity associated with the migraine attack.

## Introduction

Migraine is a common neurological disorder, causing significant socioeconomic burden [1]. This is no less true in children, where migraine can be a major cause of time off school and impaired academic performance [2, 3]. For several years, the presence of non-headache symptomatology outside of aura accompanying migraine has been noted, particularly in children, with the recognition of the episodic syndromes which may be associated with migraine [4]. These syndromes comprise benign paroxysmal torticollis [5], benign paroxysmal vertigo [6], cyclical vomiting syndrome [7] and abdominal migraine [8], as well as infantile colic [9], which has recently been added to the International Classification of Headache Disorders-3 beta in the appendix [4]. These have alluded to migraine effects outside of the brain, and their recognition has broadened the heterogeneity of the phenotype of paediatric migraine. Much of this symptomatic diversity is likely mediated by the multiple genetic and epigenetic factors involved in most migraine [10], aside from the rare monogenic forms [11].

As well as these syndromes, premonitory symptoms in migraine, defined as symptoms occurring prior to the onset of headache but excluding aura, have become an increasingly recognised area within migraine research [12]. In adults several, mainly retrospective, studies have looked at estimating the prevalence of these symptoms and their ability to warn of an impending headache, as well as their clinical phenotype and symptomatic heterogeneity [13–21]. Pivotal neuroimaging studies in 2014 captured this phase of the migraine attack for the first time using positron emission tomography (PET) [22•, 23•, 24]. Such studies are lacking in children and adolescents and so far we were only able to identify two studies looking at premonitory symptoms in the paediatric and adolescent population [25, 26••]. Characterisation of such symptoms in children can be challenging, because of the difficulties in history taking, differences in communication and cognition and the physical display of symptoms being somewhat different compared to adults.

Increased attention is required to identify such symptoms in children, as their recognition may allow early and effective attack management, further understanding of the wider impact of the migraine attack aside from pain, and improve understanding of their neurobiology, allowing future targeted abortive therapeutics research. This review will summarise our understanding to date of this early phase of a migraine attack, both from adult and paediatric studies, with the aim to increase understanding about the presence of these symptoms in children and adolescents and to enhance further interest in performing studies into these symptoms and their neurobiological basis.

### What We Have Learned about Premonitory Symptoms from Adult Studies and Translation into the Paediatric Population

#### Prevalence

Population-based and diary studies in adults have reported a range of varying prevalances of premonitory symptoms of between 9 and 88% [13–21]. There is likely a reporting bias involved, as well as difficulties in patients recognising these symptoms as being related to a migraine attack, particularly when many of them are non-specific. These factors may lead to the wide variability in the prevalences reported, as well as differences in data collection and study design (retrospective vs. prospective). The only real prospective study was performed by Giffin et al. in 2003 [14]. Prevalence could not be deduced from this study, as patients were preselected as experiencing premonitory symptoms during spontaneous attacks.

In the authors’ clinical experience, if probed the vast majority of patients will report at least some symptoms occurring before the onset of migraine pain, and often they have not recognised these as being associated with the attack, given the non-specific nature of many of the symptoms. Commonly these symptoms are mistaken for migraine triggers; such as premonitory light sensitivity being mistaken as bright light triggering a headache [27]. Additionally, a collateral history where possible from family members or colleagues can be useful; as mood change and subtle changes in concentration are usually best picked up by others.

Asking about these symptoms in children and adolescents in our paediatric headache clinic over the last few years, has also been revealing that such symptoms can be displayed even by young children, and when parents are asked, they can admit to having observed them. From the two studies of premonitory symptoms in the literature in children and adolescents, a population prevalence of premonitory symptoms cannot be deduced. Cuvellier et al. performed a clinic-based study in 103 patients in 2009 and found 67% of patients retrospectively reported at least one premonitory symptom in association with migraine [25]. In our study, patients were preselected as reporting at least one premonitory symptom and we found that two or more premonitory symptoms were reported by 85% of 100 patients in a clinic-based cohort [26••].

Given the wide range of prevalances reported in the adult literature, it is difficult to extrapolate whether the paediatric rates are compatible. Indeed, it does suggest that as physicians we should be asking about them more, both in the adult and paediatric setting. Prospective studies in the wider population would be valuable in providing a clearer understanding about the population prevalence.

### Phenotype

Although recently interest into this field has grown, non-painful symptomatology associated with migraine has been noted as far back as the nineteenth century by Gowers [28]. Despite knowledge of the existence of premonitory symptomatology for over a century, the neurobiological basis for these symptoms remains poorly understood. Symptoms can be broadly divided into categories such as sensory sensitivities: photophobia, phonophobia, osmophobia and allodynia; mood, sleep and cognitive change: low mood, fatigue, yawning, elation, irritability, poor memory and concentration difficulty; homeostatic alterations: frequency of micturition, change in bowel habit, thirst and cravings, as well as other migrainous symptoms: mild head discomfort, neck discomfort and eye discomfort.

From the adult studies performed over three decades, the phenotype of symptoms reported has been largely consistent. Additionally, the reproducibility of these symptoms across different migraine attacks [15], and their ability to predict an impending headache are also high [14]. In general, the most common symptoms seem to be mood change (anxiety, irritability, depression or elation), yawning, concentration difficulty, tiredness and neck stiffness.

The phenotype to our knowledge has only been studied in children in two studies [25]. The Cuvellier study reported face changes, fatigue and irritability as the most common symptoms [25]. Our study found fatigue, mood change and neck stiffness to be the most common [26••]. Face changes have been less frequently reported in adults [14, 29]. The reasons for the differences between the studies in the adult and paediatric populations may arise from parents and teachers looking at children more closely compared to adults self-reporting facial changes. We can expect that the reporting of premonitory symptomatology is going to be different in the paediatric population, depending on age and cognition, and because of dependence on a third party history and assessment for the symptoms through a parent, teacher or carer. Additionally, features such as face changes may be caused by pallor or facial swelling, flushing or sweating, which we would interpret as cranial autonomic features [30••]. There is of course the possibility that face changes could be related to irritability and low mood.

**Fig. 1:**
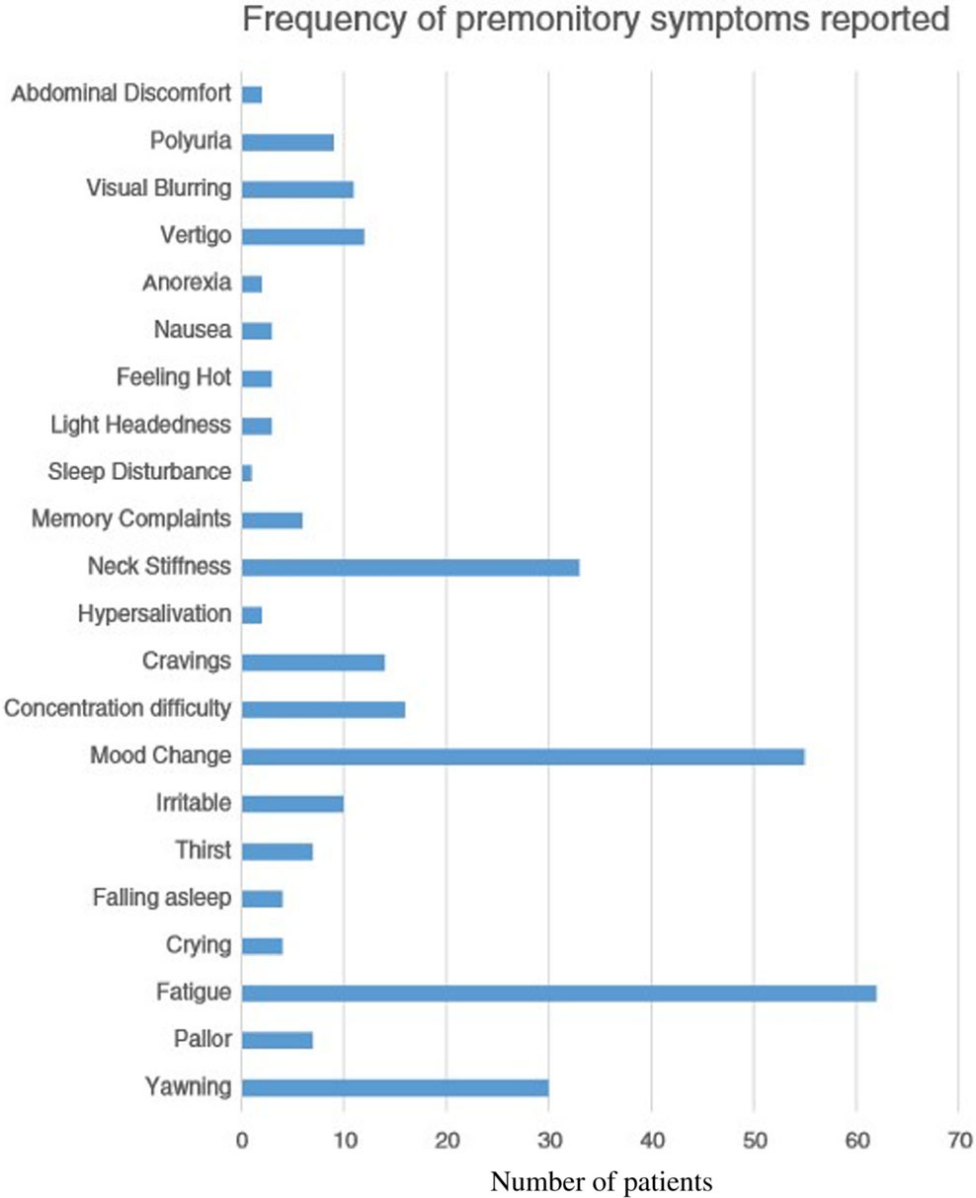
Frequency of different premonitory symptoms reported in Karsan et al. [26••] study looking at premonitory symptoms in 100 clinic patients. Courtesy of Karsan et al. [26••]

It is clear that further studies, ideally larger prospective cohort studies in the community, are required to delineate the phenotype of premonitory symptomatology in children and adolescents further and to assess the ability of the symptoms to predict a headache. Finding an appropriate way to do this in the younger children is a challenge that requires more thought and attention in the future and perhaps a specially designed electronic diary system with illustrations could be used with parent help going forward. It is also prudent going forwards that we ask about cranial autonomic symptoms presenting before headache onset or during headache in children. In our clinical and experimental experience, these features can occur before the onset of pain in adults and in the paediatric study that we conducted, pallor was reported by 7%, hypersalivation by 2% and feeling hot by 3%. Although these features may not be common, their presence and presentation before pain onset is interesting and may in the future help us understand the complex pathophysiology of migraine as a disorder, see Fig. 1.

### Neurobiology

If we take the most common symptoms reported in the literature, and anecdotally from our clinical practice in adults, we can assess the possible mediation of each symptom in a migraine patient. The localisation of symptoms in a paediatric brain is harder to assess; given patient analysis of symptoms and detailed phenotyping of each symptom may be limited in an age-dependant fashion, in view of concurrent changes in brain development and maturation.

#### Yawning

**Fig. 2:**
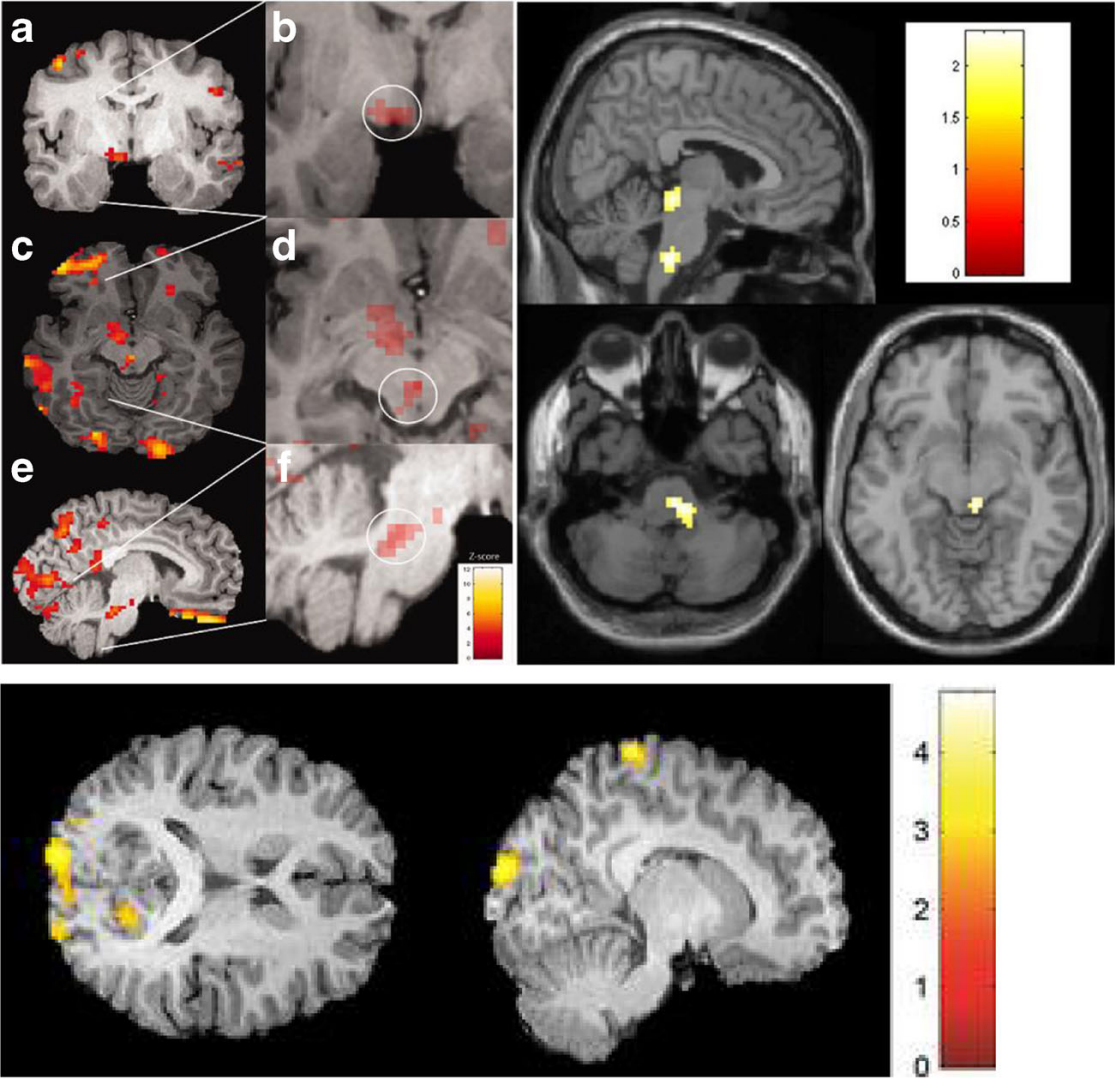
Brain areas activated during the premonitory stage of migraine attacks in the adult imaging studies. *Upper left* – Brainstem areas, including dorsolateral pons, activated, as well as periaqueductal grey and various cortical areas [22•]. *Upper right* – Nausea imaged in the premonitory phase, showing likely nucleus of tractus solitarus activation [23•]. *Bottom left* – Photophobia imaged in the premonitory phase, showing occipital cortex activation [24]. All images published with permission from the authors. Image taken with journal permissions from [23•]

Yawning is a partially dopamine-mediated symptom [31–33] and has been consistently commonly reported in adult studies of premonitory symptoms in migraine, suggesting that dopamine may be a neurotransmitter involved early in a migraine attack. Indeed small studies in the past have shown that domperidone, a selective D2 dopamine antagonist, taken during the premonitory phase may be effective at preventing headache onset [34, 35]. Of the 100 children in our study 30% reported yawning as a premonitory symptom [26••]. It was also reported in 11% of patients in the Cuvellier study [25]. The origin of this yawning in migraine may be hypothalamic, given Maniyar et al.’s imaging study showing early hypothalamic activation in a functional imaging study during the premonitory stage of migraine [22•]. See Fig. 2.

#### Mood Change and Fatigue

It is clear that chronic pain commonly causes low mood long term; but acute mood change associated with episodic acute pain conditions may not be asked about by physicians, from our experience seems unique to head pain disorders, and may be associated with cingulate gyrus activation, along with its limbic connections [22•, 36]. This is also true of fatigue associated with acute pain. In our experience from our clinic population, a large proportion of patients with migraine will report mood symptoms and fatigue throughout a migraine attack, often persisting even after headache resolution into the postdrome. These symptoms can present before the onset of pain in the premonitory phase. This is also true in children and adolescents, as both paediatric studies have shown high rates of irritability, mood change and fatigue reported [25, 26••].

These symptoms may be limbic system mediated; in particular the anterior cingulate cortex (an important part of the frontal lobe which is vital to emotional processing in pain) has also been shown to be activated during the premonitory phase [22•]. Additionally, fatigue and sleep and wakefulness disturbance may also arise from hypothalamic-brainstem connections [37]. See Fig. 2.

#### Migrainous Features

Sensory sensitivities and neck stiffness are well recognised in migraine headache, and photophobia and phonophobia are ‘migraine-defining’ in the International Classification of Headache Disorders [4]. It has been noted from the adult literature that these symptoms can start before the onset of pain; and neck stiffness was one of the most common premonitory symptoms noted in our paediatric study [26••]. Photophobia, phonophobia and osmophobia were not reported in our paediatric study and neither was allodynia. Such symptoms may be difficult to analyse for a young child. Photophobia and phonophobia were reported by small numbers in the Cuvellier study [25].

Through understanding of the neurobiology of migraine in pre-clinical models, as well as through functional imaging studies, it has become clear that sensory afferents from the dura mater and cranial circulation converge with trigeminal sensory input from the face and C2 and C3 afferents in the cord within the trigeminal nucleus caudalis (TNC) in the trigeminocervical complex (TCC), located in the brainstem. The TCC has ascending connections with other brainstem regions (periaqueductal grey (PAG), locus coereleus (LC), as well as the hypothalamus and thalamus and eventually relays nociceptive input to the cortex [38•].

The perception therefore of neck stiffness in the premonitory phase, could be through early activation of brainstem and diencephalic structures such as the hypothalamus, with loss of inhibitory input to trigeminal afferent signalling and upper cervical connections [39]. Additionally the other brainstem areas such as PAG and LC may also be active early in the premonitory phase [22•] and have similar roles in migraine in facilitating such symptoms. See Fig. 2.

Photophobia in the premonitory phase has been shown to be centrally mediated, through activation of the occipital cortex [24]. How this occipital cortex activation comes about is likely to be through ascending activation from the brainstem through the thalamus [40, 41]. Functional imaging studies have helped us develop some understanding of how early brainstem activation during the premonitory phase may cause upstream cortical activation through thalamic processing before pain has started. See Fig. 2.

Similarly a functional imaging study has helped us locate the origin of nausea in the premonitory phase [23•]. Although less commonly reported by children compared to adults (25% of adults reported nausea in the Giffin et al. study) [14], it has been another consistent symptom reported in the adult literature. The nucleus of the tractus solitarius has been implicated in experimental and imaging studies, again located within the brainstem [23•, 42]. See Fig. 2.

#### Limitations to Current Studies

As mentioned, the majority of the adult studies and both paediatric studies have involved retrospective data collection. This form of data collection leads to recall bias and questionnaires can lead to their own biases through only asking about specific symptoms recognised by the physician designing the questionnaire, whereas other symptoms may also be present. There is also the difficulty in patients being able to associate some of the non-specific symptoms with attacks, or mistaking them for triggers.

In our research environment, we have had patients observe other less well-reported symptoms in the premonitory stage when studied experimentally through exogenous triggering. These have included cranial allodynia, cranial autonomic symptoms such as nasal stuffiness and flushing, as well as facial pallor to name a few [30••]. Additionally, data analysis from retrospective studies does not allow true estimation of the population prevalence of premonitory symptoms as the study data collection populations from all the studies are so varied, and apart from the Giffin and Quintela studies, the studies do not look at the reproducibility of these symptoms across serial attacks [14, 15]. The Giffin study also was able to look at the ability of these symptoms to predict a headache attack in a prospective fashion, which the other studies have not [14].

Study designs have not been consistent throughout the literature, and the definition of premonitory symptoms has varied, which may account for some of the differences in prevalances. It is clear from the clinic that these symptoms may start before the onset of pain, or occur during the pain, and may persist after the pain has settled. It is easier to study the symptoms experimentally with imaging when no pain is present, and they are ‘true’ premonitory symptoms. However, when looking at population prevalence, the symptoms should be counted as present if they occur at any time before or during headache, as the neurobiology is likely to be the same. In our clinical practice, we have observed that patients will often deny premonitory symptoms but report concentration change, fatigue, mood change, neck stiffness and yawning during the headache phase. Whether these symptoms are disregarded before the onset of pain or only occur during headache in some patients is an area which is poorly understood. We suggest that the headache classification should include a section on premonitory symptoms and perhaps define these symptoms as ‘premonitory-like’; in that they are non-migraine defining, non-headache symptoms which can occur before or during the headache [4]. In the paediatric population, such limitations have even more impact as phenotypic descriptions of symptoms may be more varied. On considering study design for future studies or therapeutic trials, this is something to be considered.

#### Scope for the Future

Ideally, as physicians, we need to better recognise the premonitory symptoms of migraine in children and adults and ask about them diligently during our consultations, in a structured fashion. In particular asking about such symptoms occurring at any point during an attack; and warning patients where appropriate, parents and family members and teachers, to look out for such symptoms. Particularly in children who may not be able to communicate pain or other symptoms as well as adults, early recognition of symptoms may lead to better attack management and reduced morbidity, time off school and reduced impact on academic performance. Additionally, asking specifically about cranial autonomic symptoms is of value in trying to understand the mechanisms behind premonitory symptoms and the association with pain generation within the trigeminovascular system.

It is also clear that decades of work have culminated in the current theory that the brainstem is the pivotal area in migraine generation [43] and pilot imaging studies during the premonitory phase in adults have shown it to be involved early and have also hypothesised the involvement of particular, mainly subcortical brain areas, during the premonitory phase which may lead to a loss of inhibition over other trigeminal nociceptive areas and the perception of pain [22•, 23•, 24]. How this loss of inhibition comes about and whether there is a threshold effect as to whether migraine headache ensues after premonitory symptoms (we have observed premonitory-like symptoms without subsequent headache development in an experimental human migraine model) [30••] is an area which requires further work.

Particularly in the paediatric population, prospective diaries designed for children to help document premonitory symptoms would be an important tool to allow phenotypic description and population prevalence estimation of such symptoms within this population. The two studies published are limited by both being conducted in a retrospective fashion within a likely biased clinic population [25, 26••]. A community cohort study conducted prospectively with a large number of patients would be a valuable tool in understanding this area more. Additionally, where possible, functional brain imaging studies both in adults and in the slightly older paediatric population, with larger patient numbers, would further help understanding of the differences between paediatric and adult migraine and help design future therapeutics research and clinical trials.

## Conclusions

We have outlined in this review what we know so far about premonitory symptoms, how we can translate these findings into the paediatric population, where there is a paucity of studies into the premonitory phase of migraine, and have suggested areas for further work.

This is an interesting and exciting time for migraine research, with the discovery of drug agents targeted against specific mechanisms, such as those for calcitonin gene-related peptide (CGRP) [44] and the serotonin 5-HT_1F_ receptor [45], which are now in late phase clinical trials. For the first time since the triptans, there may be effective abortive drug agents available for those patients where triptans do not work or are contraindicated. Development of effective therapy for the management of acute migraine attacks is important, particularly in children where triptan licensing is limited. Further studies in the future about the role of these novel agents in the premonitory phase before the onset of pain may lead to the prevention of pain development. Clinical drug trials are limited in children but expanding the understanding of symptoms experienced, areas of brain involved, possible neurochemical systems at play and potential drug targets and pharmaceutical development is likely to have a substantial long-term impact on paediatric practice.
